# Evaluation of HER2Neu Status in Periampullary Cancers in Tertiary Care Centre in Northern India: A Three-Year Retrospective Study

**DOI:** 10.7759/cureus.46646

**Published:** 2023-10-07

**Authors:** Sumaira Qayoom, Apoorva Agarwal, Preeti Agarwal, Akshay Anand, Rashmi Raj, Sameer Gupta, Ajay Singh

**Affiliations:** 1 Pathology, King George's Medical University, Lucknow, IND; 2 Surgery, King George's Medical University, Lucknow, IND; 3 Surgical Oncology, King George's Medical University, Lucknow, IND

**Keywords:** fluorescent in situ hybridization, immunohistochemistry, her2neu, carcinoma, periampullary

## Abstract

Background

Periampullary carcinoma is a heterogeneous group of malignancies, and despite advances in treatment, its mortality rate remains high. A better understanding of the disease and factors influencing its course and potential therapeutic targets is imperative for improving its overall outcome. Through comprehensive cytogenetic analysis, it has been established that the development of periampullary carcinogenesis involves specific chromosomal aberrations, dysregulation of oncogenes, and suppression of genes in a multistep progressive manner. Our study aimed to evaluate the expression of human epidermal growth factor (HER2Neu) in periampullary cancers using immunohistochemistry and fluorescent in situ hybridization.

Material and methods

This was a retrospective study in which all consecutive cases of periampullary carcinoma diagnosed over a period of three years were evaluated. HER2neu expression was analyzed using immunohistochemistry (IHC) and fluorescent in-situ hybridization (FISH). Histopathological evaluation was performed according to the College of American Pathologists (CAP) protocol.

Results

Twenty patients were diagnosed during the study period. On histomorphologic analysis, most cases (n=17) were diagnosed as well-differentiated adenocarcinomas, the most common subsite being the ampulla of Vater and pathological staging as pT2N0Mx. On IHC, no overexpression of HER2Neu was reported in any case, but FISH analysis revealed one point of amplification with HER/centromere enumerator probe (CEP) ratio>2.

Conclusion

HER2Neu evaluation in periampullary carcinoma has limited value; thus, it could have a restricted therapeutic role.

## Introduction

The incidence of periampullary carcinoma is low, contributing to 0.5 to 2% of gastrointestinal malignancies [1]. It is a heterogeneous group of malignancies comprising tumors arising from the head of the pancreas, distal bile duct, ampulla of Vater, and duodenum. These tumors are grouped as periampullary carcinomas because they are difficult to differentiate clinically. On resection specimen examination, 60% originate in the head of the pancreas, 20% in the ampulla of Vater, 10% in the distal common bile duct, and 10% in the duodenum [2]. Pancreatic cancer contributes the most to this group of cancers, and despite advances in treatment, it has a very high mortality rate. Thus, there is a need to identify biomarkers that can serve as therapeutic targets. Human epidermal growth factor (HER2Neu) has been pivotal in breast cancer management and has been included in the treatment paradigm for gastric cancers. Several studies have shown a promising role of HER2Neu in other malignancies, such as lung, colon, and gall bladder. 

Similar to other gastrointestinal epithelial tumors, the development of periampullary carcinoma is a multistep process that encompasses various proto-oncogenes and tumor suppressor genes. Comprehensive cytogenetic analyses have demonstrated that pancreatic carcinogenesis follows a gradual multistep progressive course, characterized by specific chromosomal aberrations and abnormal activation or suppression of oncogenes and suppressor genes, such as Ki-ras2 Kirsten rat sarcoma viral oncogene homolog (K-RAS) or P53 intragenic point mutations, respectively [3,4]. Additionally, amplification leads to the activation of oncogenes such as C-myc and HER2Neu [5].

The incidence of HER2Neu overexpression in periampullary cancers has been variably reported (7-82%) in the published literature [6,7,8,9]. The variation in HER2Neu expression may be due to differences in tumor location, histological subtype, and evaluation techniques.

The paucity of information in the published literature on the Indian population encouraged us to study HER2Neu status in periampullary carcinoma for personalized treatment. In this study, HER2Neu was evaluated in periampullary carcinoma resection specimens using fluorescence in situ hybridization (FISH) and immunohistochemistry (IHC).

## Materials and methods

This retrospective cross-sectional study was performed in the Department of Pathology, King George Medical University on resected specimens of periampullary carcinomas, including pancreatic ductal carcinomas. Cases with insufficient tumor tissue for IHC and FISH were excluded from the study. We included 20 patients with periampullary carcinoma who underwent Whipple’s procedure between 2020 and 2022. After approval by the King George Medical University Clinical Research Ethics Committee (No. 1105/Ethics/2022), clinicopathological data were retrieved from the archival files. The paraffin blocks and slides were reviewed. IHC and FISH were performed on 3-4μ-thick paraffin sections of the primary tumor. In our department, resection specimens were fixed in 10% neutral buffered formalin for 18-24 hours.

Evaluation of hematoxylin and eosin (H&E) sections

H&E sections were evaluated for morphological features such as histological type, subtype, microscopic tumor extension, lymphovascular and perineural invasion, necrosis, positive/negative status of the resection margin, and lymph nodal status.

Immunohistochemistry

HER2Neu status was analyzed using IHC (clone; e2-4001+3B5, mouse monoclonal, dilution; 1/400, antigen retrieval; citrate, incubation period; 30 min, Thermo Fisher Scientific, Fremont, USA). IHC staining was performed manually. Appropriate negative and positive controls were used.

Interpretation of the HER2Neu results

In our study, we assessed HER2Neu expression using the modified 2018 American Society of Clinical Oncology (ASCO)/ College of American Pathologists (CAP) guidelines on HER2Neu testing in breast cancer via standardized immunohistochemical (IHC) testing algorithms. Membranous staining was deemed significant and scores were assigned based on the extent and intensity of staining observed in the tumor cells. Scores of 0 and 1+ were classified as negative, whereas scores of 3+ were considered positive. Score 2+ was labeled as “equivocal” and FISH testing is recommended to precisely determine HER2Neu gene status for cases in this category. However, as one of the objectives of our study was to determine the concordance between IHC and FISH results, FISH analysis was performed in all cases, regardless of the IHC score.

Fluorescent in-situ hybridization

To identify HER2Neu amplification status, the Zytolight SPEC ERBB2/CEN17 Dual color probe kit (Z-2020-20) was used per our laboratory’s standard protocol. In each primary tumor sample, at least 20 distinct and morphologically optimal nuclei were evaluated and counted. Gene amplification was considered present if the ratio between the green and red signals was ≥ 2. Evaluable signals within intact nuclei appear as separate distinct signals. Chromosome 17 polysomy can lead to false-positive results, and its identification and evaluation should be considered. If ≥ 3 red CEP17 signals were detected in > 6% of tumor cells, this was a chromosome 17 polysomy. To ensure the reliability of the hybridization results, staining intensities of sections prepared from positive and negative controls provided with the kit were comparatively evaluated. 

## Results

**Figure 1:**
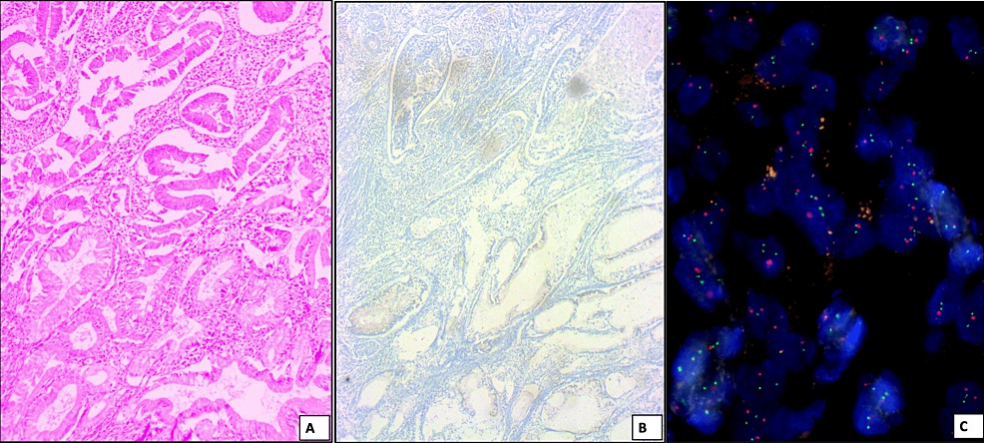
(A) A case of well-differentiated adenocarcinoma (H&E, 10X) which was negative for HER2Neu overexpression on immunohistochemistry (B) and non-amplified on FISH. (C) The red color signal represents CEP17 and the green color signal represents HER2Neu.

**Figure 2:**
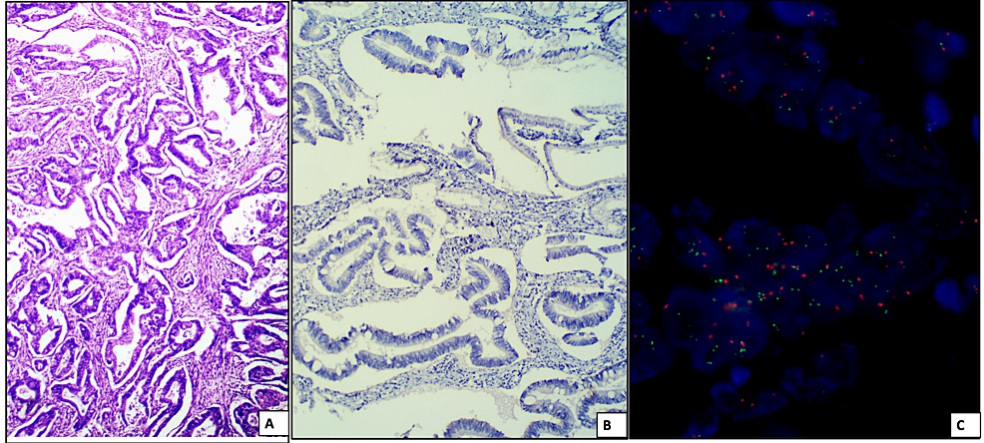
A case of well-differentiated adenocarcinoma (A)(H&E, 10X) which did not show any overexpression of HER2Neu on immunohistochemistry (B) but showed HER2Neu amplification on FISH with HER2Neu/CEP ration of 2.26 (C) Red color signal represents CEP17 and green color signal represents HER2Neu.

This study was performed on 20 patients, revealing a male predominance of 75% ( n=15), and the remaining 25% (n=5) were female. Their ages ranged from 25 to 71 years, with a mean of 50 years. On gross examination, in the majority of cases, the tumor was located in the ampulla of Vater (75%) (n=15), with the common bile duct being the second most common site (15%) (n=3) and the duodenum being the third most common site (10%) (n=2). The maximum tumor size ranged from 1 to 5 cm, with a mean dimension of 2.16 cm. On histomorphology, most cases were diagnosed as well-differentiated adenocarcinoma (n=17) (85%), and 15% (n=3) of cases were diagnosed as moderately differentiated adenocarcinoma. Out of 20 cases, 12(70%) were of the pancreaticobiliary type and eight ( 30%) were of the intestinal type. The majority of cases showed invasion into the muscularis propria of the duodenum (n=13) (65%), while few infiltrated the pancreas up to 0.5 cm (n=3) (15%) and few infiltrated the pancreas exceeding 0.5 cm in depth (n=1) (5%) and into peripancreatic tissue (n=2) (10%) as well. A single case showed infiltration into the submucosal layer of the duodenum (n=1) (5%). Perineural invasion was evident in a single case involving the pancreas (> 0.5 cm). Lymph node involvement was observed in six cases, with five cases demonstrating single lymph node involvement, while one case had two lymph nodes positive for tumor deposits. Nine cases were of pT_2_N_0_Mx, four cases of pT2N1Mx stage, three cases of pT3aN0Mx stage, and two cases each of pT3bN0Mx and pT3bN1Mx stage. The clinical and pathological features are summarized in Table 1. None of the cases showed positive staining with HER2Neu on immunohistochemistry and the score was ‘0’ in all 20 cases (Figure 1). Only a single case showed amplification with HER2Neu/CEP ratio > 2 being 2.26 on FISH analysis (Figure 2) and on repeating immunohistochemistry, it was negative for HER2Neu with a score of ‘0’. We did not find any morphological correlation with FISH amplification as a moderately differentiated tumor with pathological staging of pT3, and lymph node-positive tumors did not show immunopositivity or FISH amplification. A single case that showed amplification on FISH was diagnosed as a well-differentiated tumor that invaded the duodenum until the muscular layer only, with no lymph node involvement. On follow-up of median follow-up of 12 months, all the patients were alive until date, four of the 20 patients did not receive any chemotherapy. Two out of four were not advised chemotherapy and the remaining two patients did not receive chemotherapy despite the doctor’s recommendation and were alive to date but were severely sick, and their pathological stage was pT3bN0 and pT2N1. Chemotherapy comprised gemcitabine and cisplatin (six to eight cycles depending on the stage).

**Table 1:** Clinicopathological profile of pancreaticobiliary carcinoma cases of our study

Number		20
Mean Age		50
Sex	Male	15
	Female	05
Mean tumor size		2.16 cm
Tumor site	Ampulla of Vater	15
	Common Bile duct	03
	Duodenum	02
Tumor grade	Well differentiated	17
	Moderately differentiated	03
	Poorly differentiated	00
Histological subtype	Pancreaticobiliary type	12
	Intestinal type	08
TNM Stage:	pT_2_N_0_M_x_	9
	pT_2_N_1_M_x_	4
	pT_3a_N_0_M_x_	3
	pT_3b_N_0_M_x_	2
	pT_3b_N_1_M_x_	2

## Discussion

Periampullary carcinomas are defined as neoplasms that arise within 2 cm of the ampulla of Vater and belong to a heterogeneous group of neoplasms. On histopathology, they are subclassified as intestinal or pancreaticobiliary subtypes. Even though clinical presentation and treatment options are the same for both, the prognosis of pancreaticobiliary cancer is poor. The mainstay of treatment is surgical resection and chemotherapy with gemcitabine and cisplatin. However, given its high mortality rate, there is a need for novel therapeutic drugs. HER2Neu is an emerging biomarker in several carcinomas other than breast cancer, such as lung, esophagus, ovary endometrium, stomach, and colorectal cancer [10]. Several studies on gallbladder and pancreaticobiliary cancers have reported inconsistent results (Table 2).

**Table 2:** Summary of the published data of HER2Neu evaluation in pancreaticobiliary cancer.

Author	Sample Size	Tumor site /type	Overexpression on IHC	Amplification on FISH
Safran et al. [16]	154	Pancreatic adenocarcinoma	21%	1.9%
Stoecklein NH et al. [14]	50	Pancreatic adenocarcinoma	10%	24%
Hansel et al. [18]	32	Pancreatic adenocarcinoma	-	12.5%
Tsiambas et al. [15]	50	Pancreatic adenocarcinoma	8%	16%
Sharif et al. [19]	63	Pancreatic adenocarcinoma	25.4%	-
Komoto et al. [20]	129	Pancreatic adenocarcinoma	61.2%	-
Harder et al. [22]	207	Pancreatic adenocarcinoma	26%	3.9%
Chou et al. [17]	469	Pancreatic adenocarcinoma	7.2%	2.1%
Puhalla et al. [23]	53	Gall bladder adenocarcinoma	13%	-
Yoshikawa et al. [24]	236	Cholangiocarcinoma	12%	-
Harder et al. [25]	37	Biliary tract cancer	-	5% (genomic amplification)
Roa et al. [26]	187	Gall bladder adenocarcinoma	12.8%	-
Pignochino et al. [27]	49	Biliary tract cancer and gallbladder	-	10% (genomic amplification)
Shafizadeh et al. [28]	51	Biliary tract cancer and gallbladder	4%	-
Our study	20	Periampullary carcinoma	Nil	5%

In our study, we assessed the morphological parameters of 20 resected cases of periampullary cancer and their HER2Neu status. We found only one case with amplification on FISH, and no case showed immunopositivity on immunohistochemistry. The discrepancy between FISH and IHC positivity can be explained by the varying sensitivity and specificity of commercially available antibodies, tissue processing, methodological differences, and manual errors [11,12]. FISH techniques are more advanced and can detect specific molecular deregulatory mechanisms (intragenic mutations) apart from gene alterations, such as amplification (double minute type) responsible for protein overexpression, resulting in positive results on FISH, even in cases with 1+ or 2+ expression on IHC [13]. We compared our results with those of other studies, and similar to our findings, Stoecklein et al. [14] and Tsiambas et al. [15] reported more positive cases using FISH than IHC. However, Safran et al. [16] and Chou et al. [17] found more cases positive by IHC than by FISH, as shown in Table 2. This discrepancy can be explained by including biopsy tissue in a study by Safran et al. [16] where difficulty in performing FISH on small amounts of tissue may have been encountered.

In addition to HER2Neu status, we compiled the clinical and pathological parameters in Table 1. The mean age of our study group was 50 years, with a male preponderance of 75%, similar to studies were done by Hansel et al. [18] and Safran et al. [16] who reported a mean age of 66 years with a male preponderance of 53% [18,16]. The mean tumor diameter in our study was 2.16 cm, while Hansel et al. had 3.5 cm [18].

In our study, we did not find any significant association between high-grade tumors and HER2Neu-positive status, as our single positive case on FISH was diagnosed as a well-differentiated adenocarcinoma. Safran et al. [16] also did not find any significant association (p=0.53) between grading and HER2Neu status, as they reported HER2Neu positive status in 23% of well-differentiated cases, 16% in moderately differentiated cases, and 23% in poorly differentiated cases.

The role of HER2Neu as a prognostic marker for PDAC is controversial. Although Sharif et al. [19] and Stocklein et al. [14] found no significant association, Komoto et al. [20] found shorter survival times in patients with HER2Neu overexpression [19,14,20].

Therapies against HER2Neu receptor in patients with cancers other than breast cancer have been studied, and a complete response to lapatinib was documented in a single case of HER2Neu amplified esophageal carcinoma [21]. However, studies by Safran et al. [16] and Harder et al. [22] reported no significant survival rate in patients receiving gemcitabine and trastuzumab for pancreatic cancer.

Limitations

The limitation of our study is that due to financial constraints, we could include only a limited number of cases and studies with larger sample sizes should be conducted.

## Conclusions

Our findings indicate that while HER2Neu receptor status is positive in periampullary carcinoma, the level of positivity is not significant enough to justify initiating therapy in affected patients. Moreover, this positivity was not observed by immunohistochemistry, but only by FISH. For accurate diagnosis, we recommend combined evaluation using both IHC and FISH techniques to avoid the possibility of false negatives.
